# The frailty among suburban elderly population after one-year COVID-19 pandemic in Cirebon Regency, Indonesia

**DOI:** 10.12688/f1000research.145504.3

**Published:** 2024-09-10

**Authors:** Ahmad Fariz Malvi Zamzam Zein, Witri Pratiwi, Naswidi Dohana

**Affiliations:** 1 Department of Internal Medicine, Waled General Hospital, Cirebon, West Java, 45187, Indonesia; 2 Department of Internal Medicine, Faculty of Medicine, Universitas Swadaya Gunung Jati, Cirebon, West Java, 45132, Indonesia; 3 Department of Community Medicine and Public Health, Faculty of Medicine, Universitas Swadaya Gunung Jati, Cirebon, West Java, 45132, Indonesia; 4 Pusat Kesehatan Masyarakat Klangenan, Department of Health, The Government of Cirebon Regency, Cirebon, West Java, 45157, Indonesia

**Keywords:** Frailty, prevalence, COVID-19 pandemic, elderly, suburban population, Indonesia

## Abstract

**Background:**

The coronavirus disease 2019 (COVID-19) pandemic has had significant impacts worldwide, especially among older adults. Frailty is a determinant of susceptibility to morbidity and mortality due to COVID-19 in the elderly. This study aimed to determine frailty status and identify factors associated with the suburban elderly population in Cirebon Regency, Indonesia, after the one-year COVID-19 pandemic.

**Methods:**

A cross-sectional study of community-dwelling individuals aged ≥ 60 years was conducted in Klangenan, Cirebon Regency, Indonesia, from March to June 2021. A questionnaire was used to determine the baseline characteristics of participants, healthcare access, comorbidity, and frailty status. The Ina-FRAIL scale was used to determine the frailty status (frail/non-frail). The chi-square test and logistic regression analysis were used to determine the association between independent variables and frailty.

**Results:**

A total of 383 participants were recruited, with a median age of 67 (IQR 64-73) years. The prevalence of frailty in the present study was 10.2%. Multivariate analysis showed that age (OR 2.73; 95%CI 1.21-6.12), multimorbidity (OR 7.86; 95% CI 3.01-20.57) and financial dependence (OR 13.40, 95% CI 5.66-31.73) were significantly associated with frailty.

**Conclusion:**

One-year COVID-19 pandemic has had a considerable burden on frailty among the suburban elderly population in Indonesia. The factors associated with frailty were age, multimorbidity, and financial dependence.

## Introduction

The World Health Organization (WHO) declared the coronavirus disease 2019 (COVID-19) pandemic on 11 March 2020. ^1^ The pandemic of COVID-19 has had a tremendous impact worldwide. Studies have emphasized that people aged 60+ are categorized as high-risk groups for COVID-19 with a greater risk of severe disease, complications, and mortality. ^2^^–^^6^ During the first trimester of pandemics in Indonesia, the mortality rate of the old age population was 17.68%, which was higher than the mortality rate of young and middle-age populations (15.09%). ^5^ The mortality rate for patients more than 50 years old in Jakarta was higher (21%). ^2^ These numbers were lower than those in the US (27%) ^7^ and the UK (29%). ^8^ Furthermore, the management of the COVID-19 pandemic urged mass restrictions, promoting home confinement to reduce the spread of COVID-19. The scheme may have negative impact on elderly population’s well-being and health. ^9^ These pandemic-associated disruptive conditions necessitating robust change and adaptation that may result in increased risk of frailty. Home confinement, for example, contributes to debilitating consequences including restriction of social network and destructed continuum of care in primary health care resulting in pitfalls on medication and food issues, psychological maladaptation and increased risk of frailty. Frailty is associated with excess mortality during COVID-19 pandemic directly and indirectly. Direct pathway is due to the disease while indirect pathway is resembled by progressive decrease of physiological function and reduced resilience contributing to vulnerability and death.

Frailty is defined as a state of excess vulnerability to stressor due to age-related decline in physiologic reserve throughout multiple organ systems, resulting in inadequacy to preserve or recover homeostasis after a destabilization. ^10^ It includes social and psychological components in addition to physical dysfunction. ^11^ Behavioral adaptation as response to reduced physiologic reserve and capacity with which to meet environmental and stressor challenges leads to overt state of frailty. ^12^ Some studies showed the increase in frailty during the COVID-19 pandemic. Compared to the non-pandemic period, the COVID-19 pandemic has led to a higher risk of incident frailty among the non-frail elderly population in Japan. ^13^

The impact of the COVID-19 pandemic and its countermeasures on frailty transition has not yet been predicted, especially in Indonesia. It affects all aspects and regions in each country, not only in big cities but also in suburban regions. The impact of pandemics on suburban populations is not yet known. Thus, this study aimed to investigate the prevalence of frailty and factors associated with frailty among the elderly suburban population in Indonesia.

## Methods

### Study design and subjects

This study used data from our research project titled *The Impact of Pandemic on Elderly Adults in Cirebon* (IMPEDANCE). This cross-sectional study was conducted in a suburban community-dwelling elderly population, namely Klangenan, Cirebon Regency, Jawa Barat, Indonesia. *Badan Pusat Statistik* (Central Bureau of Statistics) Cirebon Regency estimated that there were 2,290,967 people registered at Cirebon Regency in 2021, with a total of 202,416 elderly people (8.84%). ^14^ Klangenan is a suburban region in Cirebon Regency. Of the total 53,119 people, there were 4,704 elderly people in Klangenan (8.86%). ^14^

This study was conducted between March and June 2021. We obtained a list of elderly people aged ≥60 years from *Pusat Kesehatan Masyarakat* (Public Health Center) Klangenan. We enrolled local health cadres to recruit caregivers (mostly family members) from the selected population. The inclusion criteria in this study encompassed community-dwelling elderly people with ≥60 years at Klangenan. Any unavailable contact number of caregivers was excluded.

### Study tool

The instrument used in this study was a questionnaire to determine the baseline characteristics of the participants, healthcare access, comorbidities, and frailty status. The baseline characteristics included age, sex, ethnic group, education level, occupation status, and financial dependence. The categories according to sex were male and female. Categories according to age were 60-69 years and ≥70 years. Categories according to ethnic groups were Javanese, Sundanese, and others. Categories according to educational level were elementary school or below, junior high school, senior high school, and university. The categories according to occupational status were retirement and still working. Categories based on financial dependence are dependent and independent.

Healthcare access was determined by assessing whether participants had difficulty accessing healthcare. Categories according to difficulty in healthcare access were difficult and difficult. Comorbidity was assessed by the number of comorbidities (<2 and ≥2) and the disease(s) acknowledged by participants. The categories according to the number of comorbidities were <2 and ≥2. Categories according to disease of comorbidity (ies) were diabetes mellitus (DM), hypertension, cancer, chronic obstructive pulmonary disease (COPD), coronary artery disease (CAD), dyslipidemia and stroke.

The frailty status was assessed using the Indonesian version of the FRAIL scale (Ina-FRAIL scale). It is affirmed that the utilization of the Ina-FRAIL scale in this study has been approved by copyright license permission. The FRAIL scale is one of the available valid tools used for screening and identification of frailty with high feasibility (simple) in both community and primary care settings and prognostic value in predicting frailty-related consequences (disability, geriatric syndrome, quality of life, and mortality). ^15^^–^^18^ In addition, it has been reported that the Ina-FRAIL scale is a valid (Cronbach's alpha 0.530) and reliable tool (Kappa co-efficient agreement was 0.951 (p<00.1) to screen for frailty syndrome and sarcopenia in various clinical settings in Indonesia. ^19^ It consists of five questions about fatigue, resistance, ambulatory, illness, and loss of weight with a total score of 0 to 5 for each question. Frailty status was categorized into two groups: non-frail (score 0-1) and frail (score ≥2). ^19^

### Data collection

The formulated online questionnaire was administered using a digital platform called Google Forms. The Google Forms link was disseminated by health cadres through messenger applications ( *WhatsApp*) to the caregivers. Health cadres also assisted caregivers in completing the questionnaires based on the participants’ answers. Participants’ responses were initially collected as Google Forms data, which were subsequently extracted into a spreadsheet file and exported to Microsoft Excel for cleaning and coding. The cleaned data were exported to IBM SPSS Statistics for Windows, version 26 (IBM Corp., Armonk, N.Y., USA).

### Statistical analysis

The variables were described by frequency and percentage. Any missing data will be excluded in statistical analysis. The prevalence of frailty was measured by calculating the proportion of frailty in all the subjects. Bivariate and multivariate analyses were performed in this cross-sectional study. The participants were categorized based on sex into males and females. Categories according to age group were [1] 60-69 years and [2] ≥70 years. Categories according to education level were: [1] low (elementary school or below and junior high school) and [2] high (senior high school and university). Categories according to occupation status were as follows: [1] on retirement and [2] still working. Categories according to financial dependence were [1] dependent and [2] independent. Categories according to difficulty in healthcare access were [1] difficult and [2] not difficult. Categories according to the number of comorbidities were:[1] <2 and [2] ≥2. Categories according to comorbidities were: [1] diabetes mellitus, [2] hypertension, [3] cancer, [4] chronic obstructive pulmonary disease, [5] coronary artery disease, [6] dyslipidemia and [7] stroke.

Both of bivariate analysis and multivariate analysis were performed in this study. We use chi-square test to analyse the association between variables and frailty status, with the Fisher’s exact test as the alternative test. Variables with p value <0.25 were then recruited in multivariate models using multiple logistic regression. P value <0.05 was set as statistical significance in all analysis.

### Ethical considerations

The ethical clearance had been obtained in this study. It was approved by the Health Research Ethics Committee of Gunung Jati Hospital, Cirebon City, Jawa Barat, Indonesia. The registration number is 082/LAIKETIK/KPEKRSGJ/2021 with the date of approval March 13, 2021. Submission of the answered questionnaire provided consent to participate in the study. Privacy and confidentiality were also ensured. This study adhered to the Declaration of Helsinki.

## Results

### Baseline characteristics

A total of 383 participants were male (50.4%) and female (49.6%). Most of the participants were aged 60-69 years (58.7%), with a median age of 67 years (IQR 64-73). Missing data was not identified in this study due to all participants completed the questionnaire. The baseline characteristics of the study participants are presented in Table 1. As many as 50.1% of the participants were retired and the rest were still working, including traditional traders (89.5%), farmers (5.8%), and laborers (4.7%). Most participants (91.4%) were financially independent of their businesses (76%) and pension funds (24%). More than one-fourth of the participants experienced difficulty in accessing healthcare during the COVID-19 pandemic.

**Table 1.** Demographic and disease characteristics of participants (n=383).

Characteristics	Frequency (n)	Percentage (%)
Gender		
Male	193	50.4
Female	190	49.6
Age (years)		
60–69	225	58.7
70–79	142	37.1
≥80	16	4.2
Ethnic group		
Javanese	311	81.2
Sundanese	70	18.3
Others	2	0.5
Education level		
Elementary school or below	145	37.9
Junior high school	119	31.1
Senior high school	110	28.7
University	9	2.3
Occupation status		
On retirement	192	50.1
Still working	191	49.9
Financial dependence		
Dependent	33	8.6
Independent	350	91.4
Need family caregiving		
Yes	372	97.1
No	11	2.9
Difficulty in healthcare access		
Difficult	105	27.4
Not difficult	278	72.6
Number of comorbidity (ies)		
<2	357	93.2
≥2	26	6.8
History of COVID-19		
Yes	6	1.6
No	377	98.4

**Figure 1.**
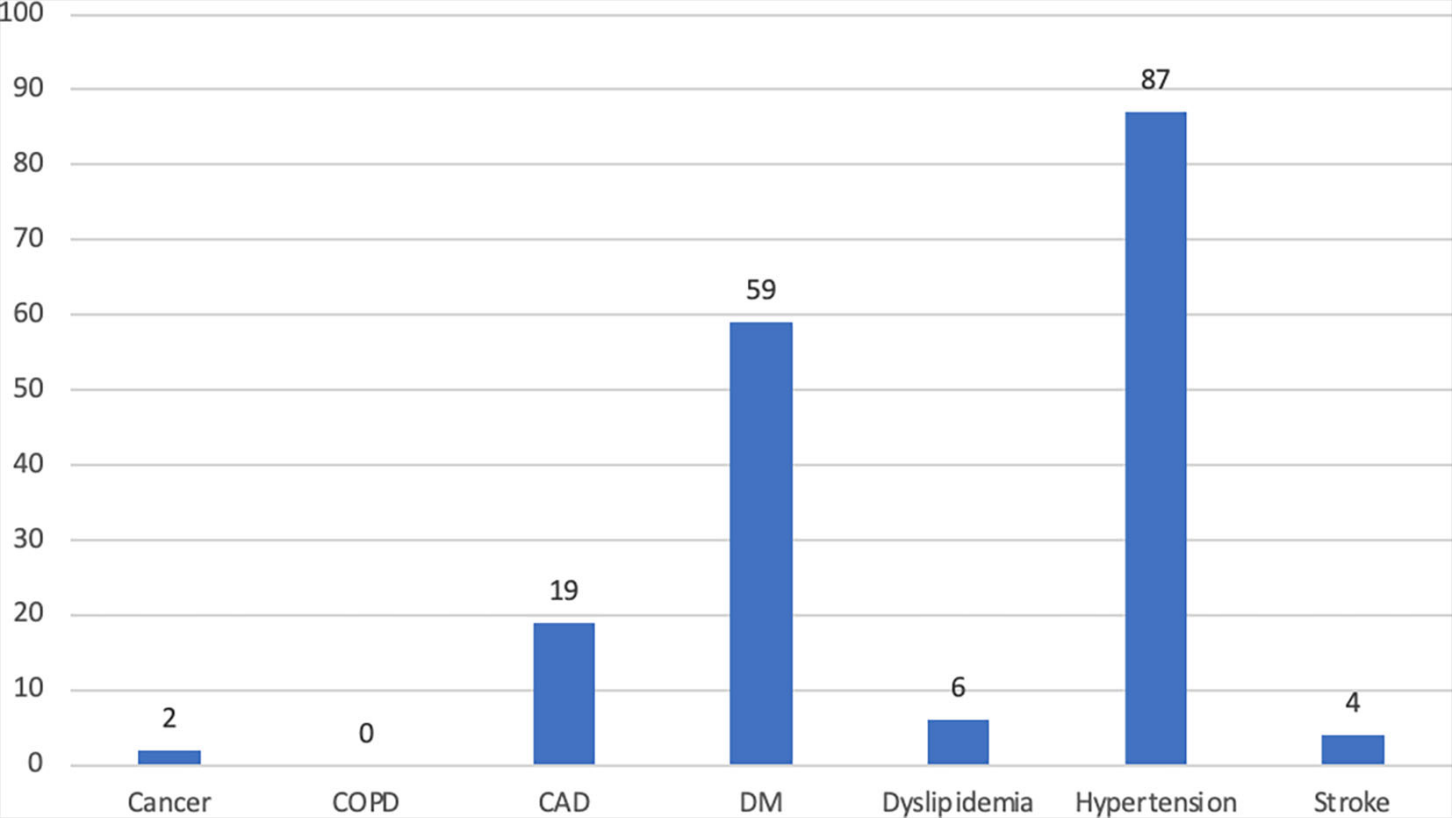
The profile of comorbidities among subjects. Abbreviations: CAD – coronary arterial disease; COPD – chronic obstructive pulmonary disease; DM – diabetes mellitus.

Comorbidity profiles are shown in Figure 1. Most participants had fewer than 2 comorbidities. Hypertension was the most frequent comorbidity (22.7%), followed by DM (15.4%), and CAD (5.0%). None of the participants were diagnosed with COPD in this study. As many as 1.6% of elderly people had been infected with COVID-19 ( Table 1).

### The characteristics of frailty

**Figure 2.**
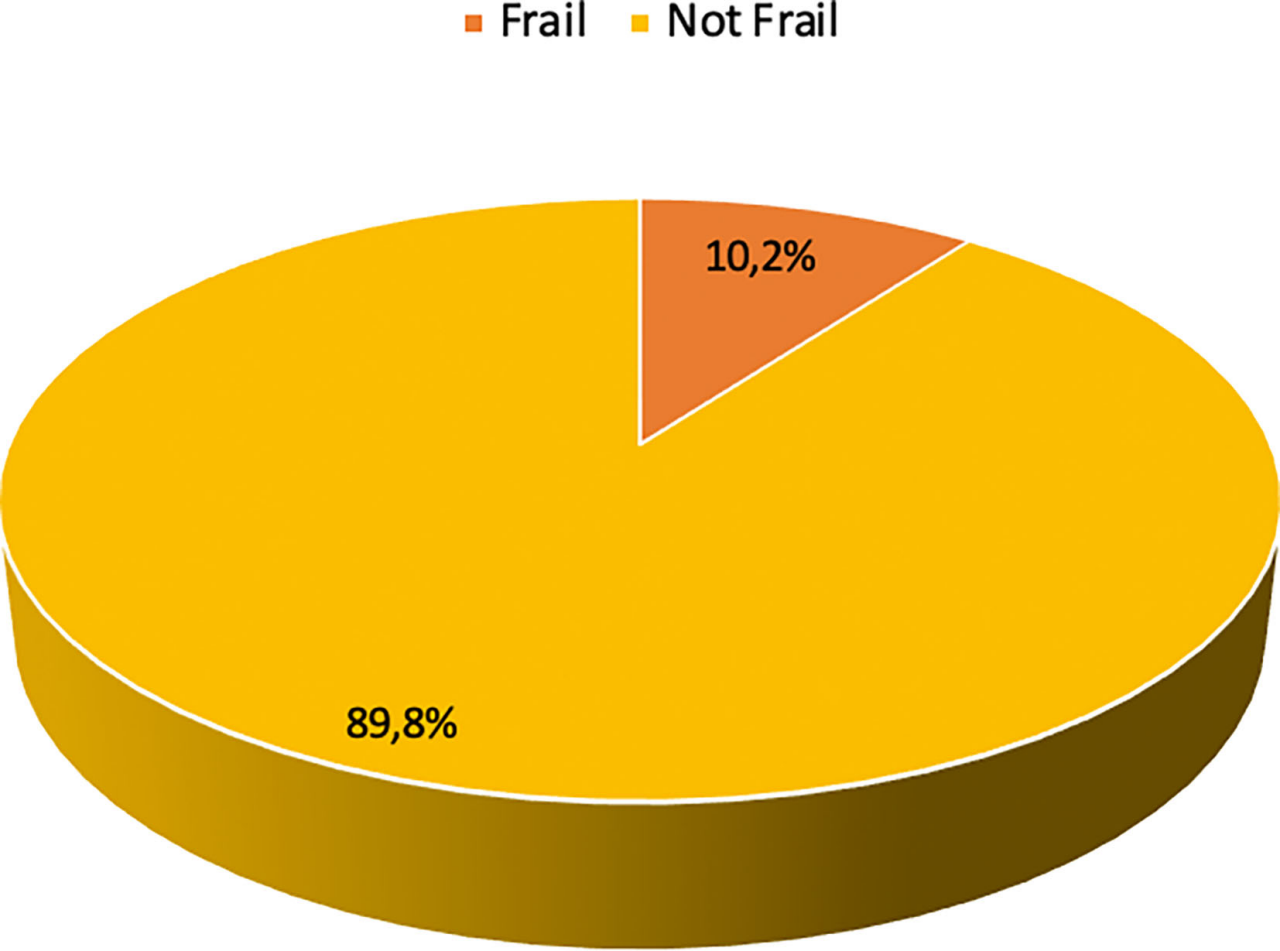
Frailty status of the participants (n=383).

The prevalence of frailty in this study was 10.2% ( Figure 2). The aspects of frailty in this study predominantly encompassed difficulty in climbing 10 stairs without rest (13.1%), difficulty in walking 100-200 meters without assistance (12.8%), and loss of weight (3.9%) ( Table 2). Only a few participants felt tired most or all of the time (0.8%) and had a considerably high burden of comorbidities (0.8%).

**Table 2.** The profile of frailty aspects.

Frailty components	Frequency (n)	Percentage (%)
Resistance		
No	333	86.9
Yes	50	13.1
Fatigue		
Seldom	245	64
Sometimes	135	35.2
Most of the time	2	0.5
All the time	1	0.3
Illnesses		
0–4	380	99.2
5–11	3	0.8
Ambulatory		
No	334	87.2
Yes	49	12.8
Loss of weight		
No	368	96.1
Yes	15	3.9

### The association between variables and frailty

Bivariate analysis revealed that some variables were associated with frailty in this study, including age (OR 3.66; 95% CI 1.79 – 7.47), education level (OR 3.37; 95% CI 1.28 – 8.85), occupation status (OR 6.40; 95% CI 2.61 – 15.67), financial dependence (OR 13.38; 95% CI 5.99 – 29.87), and number of comorbidities (OR 8.62; 95% CI 3.62 – 20.54), as shown in Table 3.

**Table 3.** The bivariate analysis.

Variables	Frailty	Not frailty	p-value	OR	95% CI
Gender					
Male	23 (11.9)	170 (88.1)	0.26	1.47	0.75 – 2.88
Female	16 (8.4)	174 (91.6)			
Age (years)					
≥70	27 (17.1)	131 (82.9)	<0.001 *	3.66	1.79 – 7.47
60–69	12 (5.3)	213 (94.7)			
Education level					
Low	34 (12.9)	230 (87.1)	0.009 *	3.37	1.28 – 8.85
High	5 (4.2)	114 (95.8)			
Occupation status					
On retirement	33 (17.2)	159 (82.8)	<0.001 *	6.40	2.61 – 15.67
Still working	6 (3.1)	185 (96.9)			
Financial dependence					
Dependent	16 (48.5)	17 (51.5)	<0.001 *	13.38	5.99 – 29.87
Independent	23 (6.6)	327 (93.4)			
Need family caregiving					
Yes	37 (9.9)	335 (90.1)	0.383	0.5	0.10 – 2.39
No	2 (18.2)	9 (81.8)			
Obstacles in accessing health services					
Yes	15 (14.3)	90 (85.7)	0.106	1.76	0.89 – 3.51
No	24 (8.6)	254 (91.4)			
Number of comorbidities					
≥2	11 (42.3)	15 (57.7)	<0.001 *	8.62	3.62 – 20.54
<2	28 (7.8)	329 (92.2)			
History of COVID-19					
Yes	2 (33.3)	4 (66.7)	0.12	0.22	0.04 – 1.23
No	37 (9.8)	340 (90.2)			

* Significant with p-value <0.05.

Furthermore, the multivariate analysis ( Table 4) demonstrated that age (odds ratio [OR] 2.73; 95% confidence interval [CI] 1.21 – 6.12), number of comorbidities (OR 7.86; 95% CI 3.01 – 20.57), and financial dependence (OR 13.40; 95% CI 5.66 – 31.73) were significantly associated with frailty among participants in this study.

**Table 4.** The multivariate analysis.

Variables	Coefficient ß	Standard error	p-value	OR (95% CI)
Age	1.002	0.41	0.015	2.73 (1.21 – 6.12)
Financial dependence	2.595	0.44	<0.001	13.40 (5.66 – 31.73)
Number of comorbidities	2.062	0.49	<0.001	7.86 (3.01 – 20.57)

## Discussion

The elder population is more vulnerable to frail during COVID-19 pandemic. Moreover, elder people with frail were less able to survive compared to those who were not frail. ^1^^,^^2^ This study revealed that frailty was considerable among older people in suburban areas, with approximately one in ten elderly people being frail. A study by Minoru Yamada et al. reported that the COVID-19 pandemic has led to a higher risk of incident frailty among the non-frail elderly population in Japan (OR 1.54, 95% CI: 1.18-2.02), with an incidence rate of 16% in year 2020-2021 vs. incidence rate of 11% in 2015-2016. ^13^ The study indicates a higher number of frail than our study. This might be due to some points: [1] the study was recruited elder population (≥65 years *vs.* ≥60years in our study) with higher mean age of participants (73.5 + 5.5 years *vs.* 68.3 + 5.9 years in our study); [2] the study was using different tool for defining frailty (the Kihon checklist). The Kihon checklist is a 25-item questionnaire that includes seven categories: daily life, physical ability, nutrition, oral condition, the extent to which one is housebound, cognitive status, and depression risk. ^20^ Other study among 593 participants in Japan by Tomoyuki Shinohara, et al., reported that the prevalence of frailty was 11.8% and there was a 3.9% increase in prevalence during the 6-month observation. ^21^ This prospective cohort study demonstrated a slightly higher prevalence of frailty in our study regarding older population in recruitment, higher mean age of participants, shorter duration of observation, and different tools in assessing frailty (using Frailty Screening Index). In addition, a cross-sectional study of 11,145 participants in the Netherlands by Sealy *et al.* revealed that frailty was present in 13% of patients during the first wave of the COVID-19 pandemic. ^22^

Frailty during the COVID-19 pandemic is a catastrophic condition that necessitates a complex and comprehensive approach to its identification and management. It is affected by, but is not limited to, age, physical stress, psychological pressure, and social detachment. Our study emphasizes that age, multimorbidity, and financial dependence play pivotal roles as factors associated with frailty in the elderly population during the pandemic.

In this study, age was significantly associated with frailty (OR 2.73, 95% CI 1.21-6.12). This is consistent with a study by Shinohara *et al*. with an OR of 1.082 (95% CI 1.050–1.115) and a study by Martine J. Sealy *et al.* with an OR of 1.04 (95% CI 1.03–1.06). ^21^^,^^22^ Age-associated decline and dysregulation may predominantly increase vulnerability. ^23^ It worsens the dysregulation of multiple interconnected physiological and biological systems exceeding a threshold to critical dysfunction homeostasis. ^23^^,^^24^ Further, age-related physiological changes, consisting of changes in body composition (decreased muscle mass), hormonal imbalances (menopause, andropause, corticopause, and somatopause), and insulin resistance can cause frailty syndrome. ^25^

Multimorbidity was associated with frailty (OR, 7.86; 95% CI 3.01.- 20.57). This association is in accordance with the results from previous studies, which were study by Tomoyuki Shinohara in Japan with OR 1.619 (95% CI 1.13-2.358) and a study by Martine J. Sealy, *et al.* with OR 1.23 (95% CI 1.16-1.30). ^21^^,^^22^ A bidirectional mechanism, rather than unidirectional pathway, between multimorbidity and frailty is suggested in a strong dose-response relationship. ^26^^,^^27^ In the presence of multiple chronic diseases, especially cardiometabolic diseases, the system failure process would be initiated by an accumulation of health deficits leading to clinical condition in the form of depletion in the physiological reserve and redundancy, which is known as frailty. ^26^^,^^28^ Regarding the burden of morbidity and mortality among patients with COVID-19, multimorbidity and frailty are associated with greater risk of severe infection COVID-19 and death. In the context of pandemic, the restriction of healthcare facilities as countermeasures during a one-year pandemic may limit healthcare coverage, including geriatric care, which leads to uncontrolled status for comorbidities among the elderly population. This may cause health deficits that may affect vulnerability and increase the risk of frailty.

Financial dependence as a low economic issue in this study was associated with frailty (OR 13.40; 95% CI 5.66-31.73). *Tim Nasional Percepatan Penanggulangan Kemiskinan* (The Indonesia’s National Team for The Acceleration of Poverty Reduction) stated that the COVID-19 pandemic has detrimental effect on Indonesia’s elderly population leading to increased risks and vulnerabilities. ^29^ It is due to limited mobility as a result of ‘stay-at-home’ policy, no access to minimum income support/pension in most elder adults and increased social exclusion and isolation contributing to depression, fears and feeling of helplessness. ^29^ These conditions further cause the elderly population more vulnerable to the economic shock of pandemic, leading to financial dependence. ^29^ Financial dependence as low socioeconomic issue disrupts older adults’ social activities, puts them away from successful social relationship and reduces their quality of life. ^30^ These direct impacts bring about the increased risk of frailty. Financial dependence as an issue in family socioeconomic status represents the individual’s ability in achieving material and social resources. ^31^ Reduced family socioeconomic status can increase frailty index and impact poor health outcome. ^31^ Further, the implementation of COVID-19 countermeasures may affect both older adults and their caregivers in unmet financial needs. This worsened their financial dependence.

This study had some limitations. The design used in this study is a cross-sectional study that limits the risk factor/cause relationship analysis due to temporal ambiguity. Moreover, the online survey format contributed to potential recall bias and was dependent on the participants’ honest responses.

The findings of this study urge physicians to comprehend the clinical and psychosocial aspects of assessing and managing frailty during a pandemic. In addition, this study recommends that the government adequately facilitates economic support among the elderly population as part of social protection during the pandemic.

Further studies with a cohort design and offline survey format are needed to investigate frailty during the pandemic and its causal relationship analysis with regard to stimulating further recommendations on healthcare for the elderly population during and after the pandemic.

## Conclusion

This study demonstrated that one-year COVID-19 pandemic has had a considerable burden on frailty among the elderly suburban population in Indonesia. The factors associated with frailty were age, number of comorbidities, and financial dependence.

### Ethical considerations

This study was approved by the Medical Research Ethics Committee of Gunung Jati Hospital, Cirebon City, Jawa Barat, Indonesia (registration no. 082/LAIKETIK/KPEKRSGJ/2021). Submission of the answered questionnaire provided consent to participate in the study. Privacy and confidentiality were also ensured.

Consent to participate: Informed consent was obtained from all individual participants in this study through their responses in the Google Form.

Consent to publish: The authors affirm that all participants provided informed consent for publication through their responses in the Google Form.

## Data Availability

Figshare. Frailty among suburban elderly population in Indonesia in one-year after pandemic. https://doi.org/10.6084/m9.figshare.25151096.v1. ^32^ This project contains the following underlying data:
- Frailty during one year after pandemic, Klangenan.xlsx (baseline data of participants) Data are available under the terms of the Creative Commons Attribution 4.0 International license (CC-BY 4.0). This project contains the following extended data:
- STROBE Checklist for Frailty Study.doc (adherence to STROBE guidelines). Figshare. STROBE Checklist for Frailty Study. https://doi.org/10.6084/m9.figshare.25151153.v1. ^33^
- Participation Information Sheet.docx. Figshare. Participation Information Sheet. https://doi.org/10.6084/m9.figshare.25194017.v1. ^34^
- Participation Consent for Publication.docx (informed consent for publication). Figshare. Participation Consent for Publication. https://doi.org/10.6084/m9.figshare.25151159.v1. ^35^
- Informed consent for participation.docx (informed consent for participation in research). Figshare. Informed consent for participation. https://doi.org/10.6084/m9.figshare.25151156.v1. ^36^ Data are available under the terms of the Creative Commons Attribution 4.0 International license (CC-BY 4.0).
