# Supplementary material for: The frailty among suburban elderly population after one-year COVID-19 pandemic in Cirebon Regency, Indonesia
Source: F1000Res. 2024 Sep 10;13:151. Originally published 2024 Mar 1. [Version 3] doi: 10.12688/f1000research.145504.3 (PMC11342023; doi:10.12688/f1000research.145504.3)
Supplement: Peer review history

# Reviewer report: Reviewer response for version 3

**DOI:** 10.5256/f1000research.171436.r322474

**Published:** 2024-09-12

**Authors:** Leni Merdawati

**Affiliations:** 1 Department of Medical-Surgical Nursing, Faculty of Nursing, Universitas Andalas, Padang, Indonesia; 2 School of Nursing, College of Nursing, Taipei Medical University, Taipei, Taiwan

The authors has made the necessary changes.

Is the work clearly and accurately presented and does it cite the current literature?

Yes

If applicable, is the statistical analysis and its interpretation appropriate?

Yes

Are all the source data underlying the results available to ensure full reproducibility?

Yes

Is the study design appropriate and is the work technically sound?

Yes

Are the conclusions drawn adequately supported by the results?

Yes

Are sufficient details of methods and analysis provided to allow replication by others?

Partly

Reviewer Expertise:

psychological problem among patients with breast cancer; return to work; COVID-19; chronic diseases.

I confirm that I have read this submission and believe that I have an appropriate level of expertise to confirm that it is of an acceptable scientific standard.

# Reviewer report: Reviewer response for version 2

**DOI:** 10.5256/f1000research.164753.r309990

**Published:** 2024-08-22

**Authors:** Ani Margawati

**Affiliations:** 1 Department of Public Health and Preventive Medicine, Universitas Diponegoro, Semarang, Central Java, Indonesia

**Brief Description**: The article titled "Previously titled: The frailty among suburban elderly population in Indonesia after one-year COVID-19 pandemic" investigates the prevalence of frailty and its associated factors in the elderly population after one year of the COVID-19 pandemic in a suburban area of Indonesia. This study is highly relevant as it sheds light on the often-overlooked suburban populations, contributing valuable insights into the impacts of the pandemic on the elderly, a particularly vulnerable group.

**Major Points:**

**Timeliness and Relevance:** The study addresses a critical and timely issue, particularly as the world continues to grapple with the ongoing effects of the COVID-19 pandemic. The focus on frailty in a suburban setting, as opposed to more frequently studied urban areas, adds to the article's significance.

 Methodological Strength: The use of a well-defined cross-sectional study design, clear objectives, and rigorous statistical analyses strengthens the reliability and validity of the findings.

**Data Validity Concerns:** A significant point of consideration is the method of data collection via Google Forms, especially given the involvement of health cadres and elderly subjects, who may have lower levels of digital literacy. This raises concerns about the validity of the data collected. If the respondents were not entirely comfortable with the digital format, it could affect the accuracy and reliability of the responses.

**Minor Points:**

**Introduction:** While the introduction is comprehensive, it could benefit from a more detailed comparison with existing studies on urban populations to better contextualize the research gap.

**Discussion:** The discussion is insightful but could be expanded to include a more in-depth exploration of the relationships between frailty, multimorbidity, and socioeconomic factors.

**Considerations for Data Collection Methodology:**

**Digital Literacy:** Given that the health cadres and the elderly subjects may not be highly familiar with using Google Forms, the potential impact on data quality should be addressed. It would be beneficial to discuss any steps taken to mitigate this issue, such as providing training to the health cadres or offering assistance during the data collection process.

**Validation Methods: **Consider discussing the validation methods used to ensure the accuracy of the data collected through Google Forms. For example, were there any follow-up interviews or checks to confirm the responses? If not, this might be an area to explore in future studies to strengthen the validity of your findings.

Is the work clearly and accurately presented and does it cite the current literature?

Yes

If applicable, is the statistical analysis and its interpretation appropriate?

Yes

Are all the source data underlying the results available to ensure full reproducibility?

Yes

Is the study design appropriate and is the work technically sound?

Yes

Are the conclusions drawn adequately supported by the results?

Yes

Are sufficient details of methods and analysis provided to allow replication by others?

Yes

Reviewer Expertise:

Nutrition Science

I confirm that I have read this submission and believe that I have an appropriate level of expertise to confirm that it is of an acceptable scientific standard.

# Reviewer report: Reviewer response for version 2

**DOI:** 10.5256/f1000research.164753.r292136

**Published:** 2024-08-05

**Authors:** Leni Merdawati

**Affiliations:** 1 Department of Medical-Surgical Nursing, Faculty of Nursing, Universitas Andalas, Padang, Indonesia; 2 School of Nursing, College of Nursing, Taipei Medical University, Taipei, Taiwan

**Introduction**

**Paragraph 1**

 The authors should consider discussing the disruptive conditions during the pandemic that pose risks of frailty among older patients. Additionally, they should explain the connection between frailty and mortality rates, particularly for patients over 50 years old, and compare these findings with data from other countries.

**Methods**

**Study design and subjects**

 The authors should explicitly state how they aggregated the 383 participants, potentially through pooling data from multiple prior projects that met inclusion criteria. If conducted, the authors should describe the method used for sample size determination. This typically involves factors such as statistical power, effect size, and population variability. Justification for choosing 383 participants should be provided based on these criteria.

**Study tool**

 Frailty status was assesses using Ina-FRAIL Scale, the authors mentioned this tool is valid and reliable, please provide the Cronbach’s alpha value.

**Results**

 Baseline characteristics

 Since the research design is cross sectional, the table legend (table 1) and sub heading result “baseline characteristics” should be replaced with demographic and disease characteristics

Is the work clearly and accurately presented and does it cite the current literature?

Yes

If applicable, is the statistical analysis and its interpretation appropriate?

Yes

Are all the source data underlying the results available to ensure full reproducibility?

Yes

Is the study design appropriate and is the work technically sound?

Yes

Are the conclusions drawn adequately supported by the results?

Yes

Are sufficient details of methods and analysis provided to allow replication by others?

Partly

Reviewer Expertise:

psychological problem among patients with breast cancer; return to work; COVID-19; chronic diseases.

I confirm that I have read this submission and believe that I have an appropriate level of expertise to confirm that it is of an acceptable scientific standard, however I have significant reservations, as outlined above.

# Response

**Published:** 2024-09-03

**Authors:** Ahmad Fariz Malvi Zamzam Zein

**Affiliations:** Internal Medicine, Universitas Swadaya Gunung Jati, Cirebon, West Java, Indonesia

We, the authors of this article, are thankful for your precious review. It is thoroughly delivered in delicate assessment and recommendation.

 As you noted the importance of discussion on disruptive condition and its pathway, the amendement has been provided as we, the authors, agreed to emphasize it as well.

 Regarding the subject recruitment, it was held thoroughly in this study project, not from pooling data or multiple projects. Sample size in this study was calculated using the Open Source Epidemiologic Statistic for Public Health website ( https:://www.openepi.com/SampleSize/SSPropor.htm). By using a population size of 202,416, outcome proportion of 16% and confidence level of 95%, the minimum sample size for this study was 207.

 We appreciate for your notification on providing the validity and the reliability of Ina-FRAIL scale used in this study. In accordance with study by Dwipa L, et al., the internal consistency coefficient (assessed using the Cronbach's alpha) of Ina-FRAIL scale was 0.530 and test-retest reliability (assessed using Kappa co-efficient agreement) with a 4-week interval was 0.951 (p<0.001). Both coefficients have been added to revised manuscript.

 We have modified the word 'baseline characteristics' in the title of Table 1 into 'demographic and disease characteristics'.

 We hope that the revised version suits your review.

 Thank you in advance.

# Reviewer report: Reviewer response for version 1

**DOI:** 10.5256/f1000research.159461.r253787

**Published:** 2024-03-28

**Authors:** Nata Pratama Hardjo Lugito

**Affiliations:** 1 Department of Internal Medicine, Faculty of Medicine, Pelita Harapan University, Tangerang, Indonesia

The article titled "The frailty among suburban elderly population in Indonesia after one-year COVID-19 pandemic" was well written.

 The result and discussion was clear and concise, yet explains the condition of frailty in suburban Indonesian elderly. 

 The exact province or region in Indonesia where the study was conducted should be added to the title, as Indonesia is very vast and each region has its special condition. 

 The authors could compare in the discussion how the COVID-19 pandemic affected frailty among elderly based on other study in Indonesia or other countries, compared to before the pandemic.

 The authors have explained that socioeconomic status influenced frailty among elderly after pandemic. The authors could elaborate on the interplay of multimorbidity as risk factors of frailty and COVID-19,

 The authors did not include history of COVID-19 infection as risk factor of frailty. Could there be any data about this?

 The authors wrote the conclusion as "This study demonstrated that one-year COVID-19 pandemic has had a considerable impact on frailty..." As the authors did not mentioned in the discussion about frailty before the pandemic, it would be difficult to conclude that the pandemic impacted elderly frailty.

Is the work clearly and accurately presented and does it cite the current literature?

Yes

If applicable, is the statistical analysis and its interpretation appropriate?

Yes

Are all the source data underlying the results available to ensure full reproducibility?

Yes

Is the study design appropriate and is the work technically sound?

Yes

Are the conclusions drawn adequately supported by the results?

Yes

Are sufficient details of methods and analysis provided to allow replication by others?

Yes

Reviewer Expertise:

Internal medicine, clinical epidemiology, infectious disease

I confirm that I have read this submission and believe that I have an appropriate level of expertise to confirm that it is of an acceptable scientific standard.

# Response

**Published:** 2024-04-07

**Authors:** Ahmad Fariz Malvi Zamzam Zein

**Affiliations:** Internal Medicine, Universitas Swadaya Gunung Jati, Cirebon, West Java, Indonesia

We, the authors of this article, are thankful for your precious review. It is thoroughly delivered in comprehensive assessment and recommendation.

 Due to your comment regarding the scope of region, we have modified the title into 'The frailty among suburban elderly population after one-year COVID-19 pandemic in Cirebon Regency, Indonesia'. As your explanation, it is notable to express the region where the study held clearly.

 Some additional statements were added to emphasize the interplay of multimorbidity, frailty and COVID-19, as your recommendation. It is available at Paragraph 4 in Discussion section.

 You recommended that the history of COVID-19 infection is included in the study, if any. We have provided the history of COVID-19 in Table 1 and adjacent description. Further, it is recruited in bivariate analysis as well (Table 2).

 We have modified the word 'impact' in conclusion into 'burden'. It is aimed to notify the proportion of frailty as the magnitude of problem without reflecting pre-post pandemic effect.

 There are some modifications and corrections in writing as integrated part of our commitment in performing improvement in this article.

 We hope that the revised version suits your review.

 Thank you in advance.
